# Dose uncertainties associated with a set density override of unknown hip prosthetic composition

**DOI:** 10.1002/acm2.12167

**Published:** 2017-08-30

**Authors:** James D. Rijken, Christopher J. Colyer

**Affiliations:** ^1^ Adelaide Radiotherapy Centre Flinders Private Hospital Bedford Park South Australia

**Keywords:** dose, implant, override, prostheses, prosthetic, uncertainty

## Abstract

The dosimetric uncertainties associated with radiotherapy through hip prostheses while overriding the implant to a set density within the TPS has not yet been reported. In this study, the uncertainty in dose within a PTV resulting from this planning choice was investigated. A set of metallic hip prosthetics (stainless steel, titanium, and two different Co‐Cr‐Mo alloys) were CT scanned in a water bath. Within the TPS, the prosthetic pieces were overridden to densities between 3 and 10 g/cm^3^ and irradiated on a linear accelerator. Measured dose maps were compared to the TPS to determine which density was most appropriate to override each metal. This was shown to be in disagreement with the reported literature values of density which was attributed to the TPS dose calculation algorithm and total mass attenuation coefficient differences in water and metal. The dose difference was then calculated for a set density override of 6 g/cm^3^ in the TPS and used to estimate the dose uncertainty beyond the prosthesis. For beams passing through an implant, the dosimetric uncertainty in regions of the PTV may be as high as 10% if the implant composition remains unknown and a set density override is used. These results highlight limitations of such assumptions and the need for careful consideration by radiation oncologist, therapist, and physics staff.

## 1 INTRODUCTION

Treatment of patients with single or bilateral hip replacements requires careful consideration due to the dose effects produced through radiation interactions with the metal prostheses. Much work has already been conducted in quantifying the dosimetric effect of metal prostheses and determining the relevant properties of materials.^1^, ^2^, ^3^, ^4^, ^5^, ^6^ A greater body of literature has been collected in AAPM's TG63 report.^7^ TG‐63 also provides clear recommendations on how to undertake accurate treatment of a patient with a hip prosthetic. In the interest of accuracy, the report concerns itself with the scenario when the hip prosthetic material is already known, either in the patient records or measured through methods suggested and subsequently demonstrated in the literature.^8^ Many centers, including Adelaide Radiotherapy Centre (ARC), do not ascertain the hip prosthetic material but instead override any hip prosthetic to a set density in the treatment planning system (TPS).

There have been many documented issues with the handling of prostheses by computed tomography (CT) including image artifacts and incorrect CT to density conversion.^9^, ^10^ Integer storage limitations in the TPS and CT scanner also influence the accuracy of the prosthetic's CT number. A CT‐electron density curve is routinely checked^11^ through a phantom measurement with varying density inserts, ranging from lung density to titanium. The titanium insert receives the greatest CT number possible (2^12^ − 1001 = 3095). Materials with density greater than titanium saturate CT number and are thus indistinguishable from each other on the image. Some 12‐bit scanners are able to perform reconstructions at higher CT numbers through increased bin sizes although this method reduces fidelity. Alternatively, a centre might make improvements to dosimetric accuracy through acquisition of a 16‐bit scanner,^10^ although a 16‐bit CT image dataset imported into a 12‐bit TPS will have higher CT numbers truncated. Even if all CT information was importable, there still be may be issues with the TPS treatment of the high density regions.^9^ Some centers have evaluated the accuracy of their dose calculation algorithm using the raw density values provided by the CT scanner without override,^9^ whereas others choose to override the prosthetic to some predetermined density — which is the chief concern of this paper. While several authors have used Monte Carlo simulations to assess the accuracy of dosimetric calculations by the TPS in proximity to metallic implants with varying results,^12^, ^13^ to our best knowledge this paper is the first to investigate dose accuracy with realistic prosthesis geometries.

Because the prosthetic material remains unknown, there is uncertainty in the dose distribution from assuming a particular override value. Presently, there is insufficient data in the literature to calculate such uncertainties. Two experiments were conducted. First, it was determined which values of density override best correspond with measurement. These were compared to values obtained from the literature. Second, the uncertainty in dose was quantified from assuming a particular override value.

## 2 MATERIALS AND METHODS

**Figure 1:**
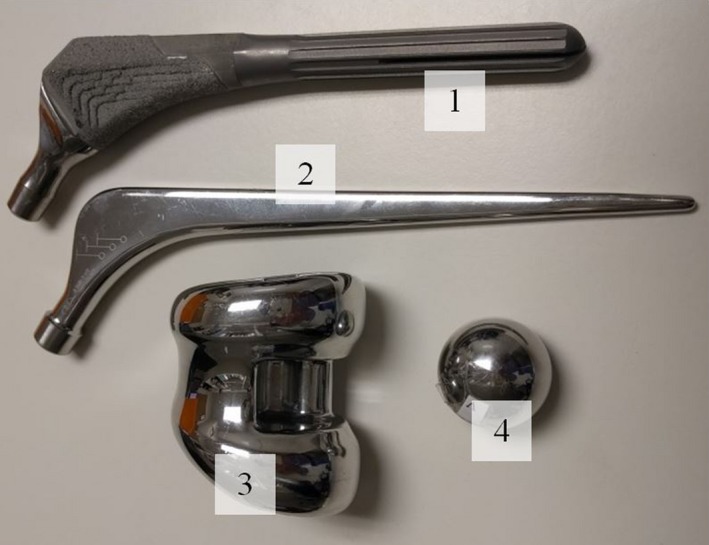
Different prosthetic pieces used. (1) titanium, (2) stainless steel, (3) CoCrMo, (4) Vitallium™.

Commercially available metal hip replacement prostheses pieces were acquired of different materials: stainless steel (SS), titanium, and two different Co‐Cr‐Mo alloys (one denoted Vitallium™ with a relative respective weight composition of 65‐30‐5). The range of relative weights of cobalt, chromium and molybdenum in alloys for prostheses are 57.4–65, 27–30, and 5–7, respectively.^4^ Stainless steel also varies in its composition. The prosthetic pieces are shown in Fig. 1 and the properties for the materials are shown in Table 1.

**Table 1:** Properties of hip replacement prosthesis alloys

Alloy	Average physical density	Mass attenuation at 4 MV (cm^2^/g)	Effective atomic number	Average electron density (e^−^/cm^3^)	Relative electron density
Stainless steel	8.1	0.047	26.7	2.3 × 10^24^	6.83
Titanium	4.3	0.044	21.4	1.2 × 10^24^	3.60
Co‐Cr‐Mo	7.9	0.048	27.6	2.2 × 10^24^	6.74

The prosthetic pieces were placed individually in a water bath and scanned on a Somatom^®^ Emotion^®^ 6 CT (Siemens AG, Munich, Germany) scanner at 100 kVp. The CT image datasets were then exported to Pinnacle^3®^ 9.8 (Koninklijke Philips N.V., Amsterdam, the Netherlands).

In the TPS, fields of 6 MV and 10 MV (respective TPR_20,10_ values of 0.680 and 0.733) were added at a gantry angle of 0°. The isocenter was set to a point 4 cm below the prosthetic. This distance is greater than the buildup distances involved and is a good representation of the distance between the femoral head and the planned target volume (PTV) (a distance of 3 cm is common for prostate‐bed patients). The streaking and image artifacts were removed by overriding the water bath to a density of 1 g/cm^3^. Contours were created for the prosthetic pieces as per their physical dimensions, to obviate issues with metal artifacts in the CT image set, particularly the concave shape of the Vitallium implant. Although vitally important to clinical implementation, the challenges of and solutions to metal artifact reduction in CT are well documented elsewhere.^14^, ^15^ The prosthetic pieces were each overridden to a series of densities ranging from 3 to 10 g/cm^3^ with a planar dose profile exported for each density and a dose point reported at depth behind the prosthetic. The dose calculations were made using the collapsed cone convolution (CCC) algorithm.

**Figure 2:**
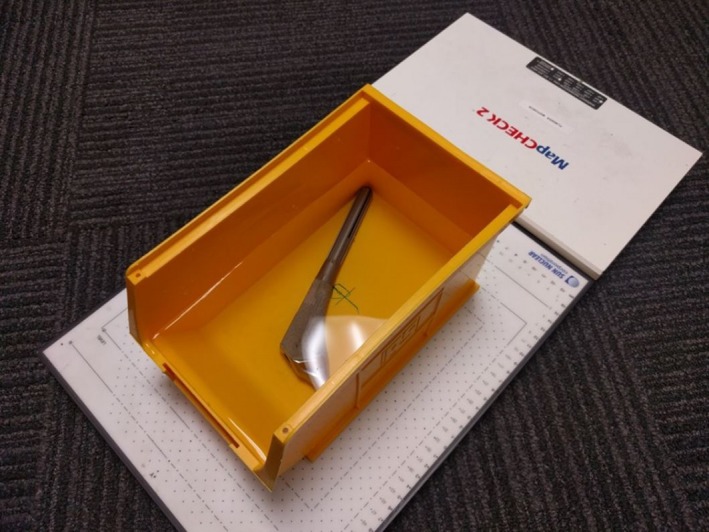
Placement of prosthetic in water bath on top of MapCheck^®^ device.

The water bath was set up on an Elekta Synergy^®^ linear accelerator (Elekta, Stockholm, Sweden) with a MapCheck^®^ 2 device underneath (Sun Nuclear Corp., Melbourne, Fl, USA) (Fig. 2). The bath was irradiated with the 6 MV and 10 MV beams for each of the four prosthetic pieces. The dose maps were acquired using SNC Patient™ 6.1 (Sun Nuclear Corp., Melbourne, FL, USA). These were compared (absolute dose mode using gamma criterion of 2%/2 mm) to the dose profiles exported by the TPS in order to determine the most accurate effective override density.

The MapCheck^®^ device was used as the higher resolution of film was not necessary for the particular purpose. Through a series of open field measurements the MapCheck^®^ has been cross‐calibrated against an ion chamber whose calibration is traceable to a primary standards laboratory and it is routinely used for absolute dose measurements of modulated treatment plans. MapCheck measurements were further supported through measurement of absolute dose using a calibrated RAZOR™ Chamber (IBA Dosimetry GmbH, Schwarzenbruck, Germany) in a solid water slab. Measured dose at a point behind the metallic implant was compared to TPS calculations using density overrides from 3 to 10 g/cm^3^ in order to find the most optimal, or effective, density override.

## 3 RESULTS AND DISCUSSION

**Figure 3:**
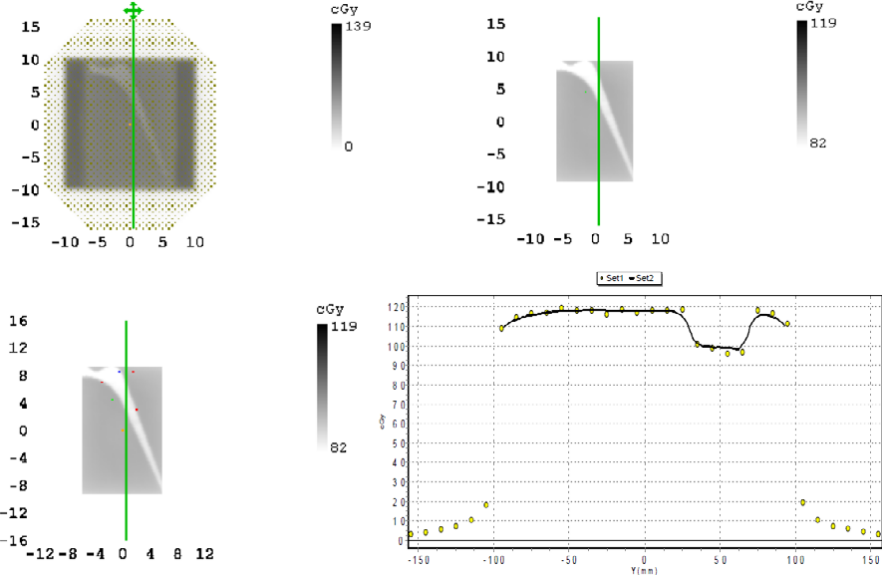
Comparison of TPS and measured dose maps for stainless steel. Clockwise from top left: measured dose map, TPS exported dose map, dose map with threshold considered and points out of tolerance (red points > 2%/2 mm), profile comparison.

**Figure 4:**
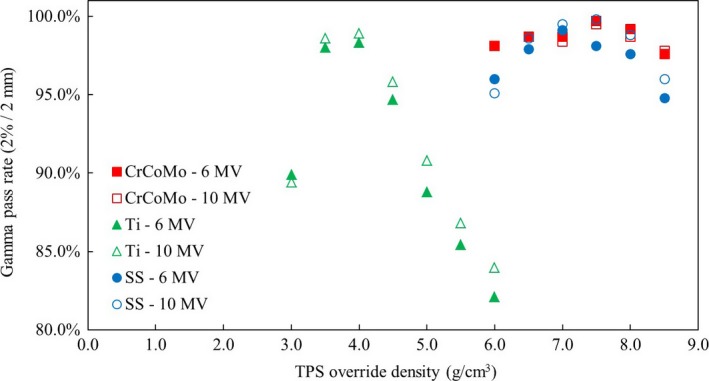
Gamma pass rates from comparisons of measured MapCheck and exported TPS dose map for a range of TPS override densities. Higher gamma pass rates indicate more optimal override density.

An example of a MapCheck measured profile and TPS export comparison is shown in Fig. 3 and a plot of gamma pass rates with TPS density override is presented in Fig. 4.

Table 2 presents the effective override densities calculated from both MapCheck and ion chamber measurements compared to data in the literature.^4^ Results from both methods and beam energies were found to agree with each other, within error.

**Table 2:** Effective override physical densities for different prosthetic materials at different beam energies in Pinnacle^3®^ 9.8. Uncertainties in effective density come from the 2.2% calibration uncertainty of the RAZOR chamber and the choice of sampling resolution of density overrides for the MapCheck

Alloy	Physical density (g/cm^3^)^4^	6 MV		10 MV	
		MapCheck (g/cm^3^)	RAZOR (g/cm^3^)	MapCheck (g/cm^3^)	RAZOR (g/cm^3^)
Stainless steel	8.1	7.0 ± 0.3	7.4 ± 0.7	7.5 ± 0.3	7.3 ± 0.8
Titanium	4.3	4.0 ± 0.3	4.4 ± 0.4	4.0 ± 0.3	4.3 ± 0.5
Co‐Cr‐Mo	7.9	7.5 ± 0.3	7.9 ± 0.7	7.5 ± 0.3	7.4 ± 0.7

It was difficult to make a hypothesis regarding the dosimetric accuracy of the TPS due to the range of results presented in the literature. Roberts^9^ showed that for a Helax TMS pencil beam algorithm the TPS calculation overdosed in the presence of steel and underdosed for titanium. He attributed the differences to the method in which the TPS assigned density from the CT, particularly its handling of saturation and artifacts, reporting a definite energy and metal density dependence for the dosimetric treatment of prosthetics. Similarly for another idealized scenario, Palleri et al.^13^ showed that the downstream dose calculations with a convolution‐type algorithm matched well with Monte Carlo models of the setup for a range of different implant materials, of the order of 3%. Conversely, a more detailed Monte Carlo simulation by Kairn et al.^12^ of spinal implant dose perturbation, again for a convolution‐type algorithm, demonstrated that the TPS was underdosing by up to 9.5% at 5 cm downstream.

Furthermore, the three studies quoted above all differed slightly from the setup in this study. The main differences in this paper include: (a) utilization of CT images for planning, to incorporate the uncertainties in the treatment planning method associated with overrides of artifacts and structures are included, (b) use of both an ion chamber and a silicon diode array which are both readily available in most clinics and can provide absolute dose information as well as dose map comparison, and (c) consideration of the uncertainties introduced by the “set override” planning choice to determine the most accurate effective override values.

This study observed that while titanium had effective density overrides that were within uncertainty of the reported values, stainless steel (and to a lesser extent, Cr‐Co‐Mo) required a lower density override in order to calculate dose accurately. This indicated that the Pinnacle^®^ CCC algorithm is more likely to underdose as physical density increases.

Like other convolution algorithms, Pinnacle^®^ CCC considers different density media to be density‐scaled water. If the familiar total mass attenuation coefficient plot is considered comparing water and lead, the coefficients for water are known to keep decreasing for energies less than 100 MeV.^16^ However, due to the strong Z^2^ dependence of the pair production interaction, the total mass attenuation coefficient for cobalt begins to increase beyond 5 MV [the pair production atomic attenuation coefficients for metals at 10 MV are indistinguishable from 6 MV nominal energies^17^]. So while Pinnacle^®^ will treat an implant as “super dense water” with the same attenuation properties, in reality the attenuation will be different due to pair production.

**Figure 5:**
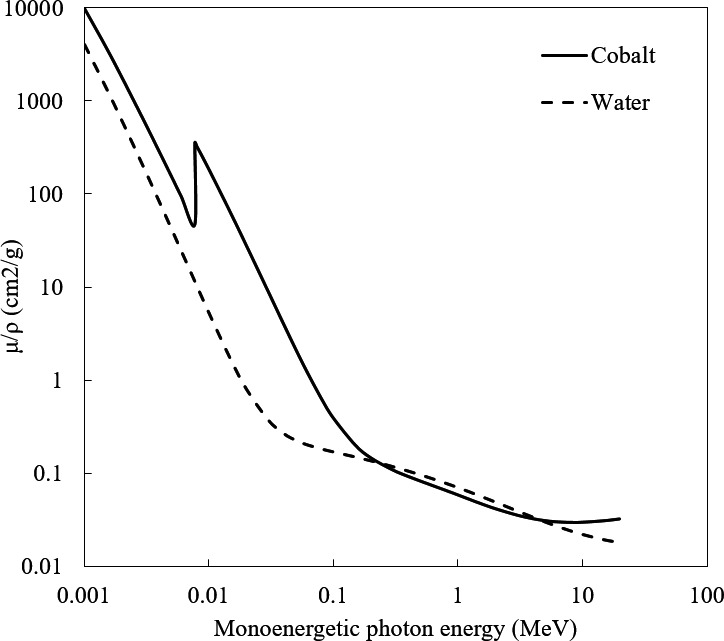
Plot of total mass attenuation coefficients for water and cobalt (18).

The underdosing observed may be due to the total mass attenuation coefficient actually being greater for water than for metal at the effective beam energies concerned. This is represented in a plot of tabulated NIST data below^18^ (Fig. 5). With an atomic number of 27, cobalt is a good surrogate for both stainless steel and Co‐Cr‐Mo (see Table 1). Then for the energies utilized, the total mass attenuation coefficient for water is around 15% greater than for Cobalt. Since Pinnacle^®^ treats everything as density‐scaled water, this is consistent with the results that the TPS may underdose behind higher atomic number alloys. This result is consistent with predictions from the Monte Carlo simulations performed by Kairn et al.^12^

**Figure 6:**
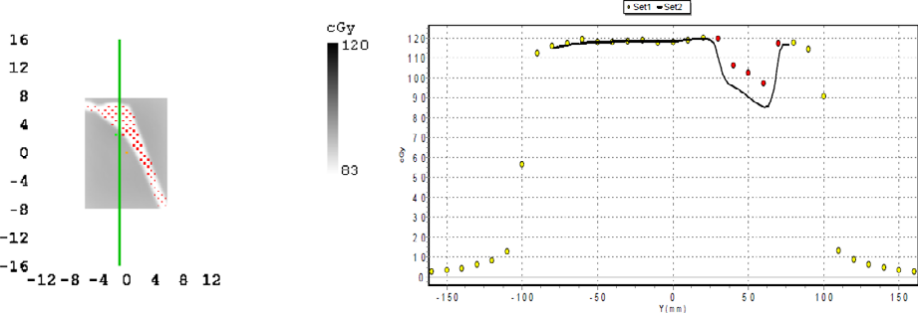
Comparison of TPS with a 6 g/cm^3^ override and measured dose maps for Ti. Left: dose map with threshold considered and points out of tolerance (red points at 2%/2 mm), right: profile comparison.

Given an ideal scenario where a patient presents with a prosthetic of known material, the appropriate density override can be selected based on material and beam energy. For situations where the prosthetic composition remains unknown, a generic override density can be used with the aim to reduce the uncertainty in the dose calculation. In order to minimize the uncertainty for any one material, a generic density of 6 g/cm^3^ was chosen as it represents the midpoint of the maximum and minimum effective override densities. The dose difference in the dose shadow was assessed for this case by dose map comparison with TPS exported profiles with a 6 g/cm^3^ override for the MapCheck and dose point comparison for the RAZOR. This is shown in Fig. 6. The dose differences and uncertainty, has been shown in Table 3.

**Table 3:** Greatest dose differences from MapCheck and RAZOR when an override of 6 g/cm^3^ is chosen. MapCheck is able to show greater dose differences because it samples many more points than the RAZOR

Alloy	6 MV dose difference		10 MV dose difference	
	MapCheck (g/cm^3^)	RAZOR (g/cm^3^)	MapCheck (g/cm^3^)	RAZOR (g/cm^3^)
Stainless steel	3.3%	4.5%	4.5%	3.2%
Titanium	−10.0%	−7.8%	−9.3%	−8.0%
Co‐Cr‐Mo	6.9%	3.4%	6.5%	1.3%
Total dose uncertainty:	+7%/−10%		+7%/−9%	

An override of 6 g/cm^3^ is denser than the most accurate effective override for titanium so the TPS will obviously underestimate dose. Similarly, 6 g/cm^3^ is less dense than the optimal overrides for stainless steel and the Co‐Cr‐Mo alloys so the TPS will overestimate dose. The dose error is not expected to be constant throughout the PTV as it will change slightly with distance from the implant.^9^ However, since the distance of 4 cm represents a point inside or close to the edge of the PTV it is a dose uncertainty of potential significance. Thus given a patient with an unknown hip prosthesis material with beams passing through the implant, the uncertainty in dose calculation at regions within the PTV may range between +7% and −10% for 6 MV and between +7% an −9% for 10 MV. These uncertainties could be clinically significant^19^ and steps should be taken by radiotherapy departments to minimize the impact of these uncertainties.

It is recommended to utilize a value of 6 g/cm^3^ for overriding hip prostheses within Pinnacle for the 6 and 10 MV beams as this value is a good compromise between the “effective” densities of Ti, stainless steel and Co‐Cr‐Mo. The very small difference in maximum dosimetric uncertainty between 6 and 10 MV as well as the very similar mean “effective” densities (Table 2) support a value of 6 g/cm^3^. This recommendation is made in the absence of presentation rates of the different implant materials. If this information was available, one may elect to push the value higher (toward stainless steel) or lower (toward Ti) to minimize the uncertainty for the most common implants.

## 4 CONCLUSION

A set of metallic hip replacement pieces were used to determine the dosimetric uncertainty associated with prostate planning protocols that involve a set override value for implants. An effective override density was found for each case that best matched the measured dose map. The dose difference was then calculated for the case where a set density override of 6 g/cm^3^ was chosen. PTV dose uncertainty in the dose shadow was found to be as much as 10%, highlighting the need for careful planning and dose consideration when allowing beams to pass through metallic implants. In instances where the composition of the metallic implant remains unknown, we recommend the use of a density override value of 6 g/cm^3^.

## ACKNOWLEDGMENTS

Thanks go to Jason Morton for the hip prosthetics.

## CONFLICT OF INTEREST

The authors declare no conflict of interest.

## References

[1] Sibata CH , Mota HC , Higgins PD , Gaisser D , Saxton JP , Shin KH . Influence of hip prostheses on high energy photon distribution. Int J Radiat Oncol Biol Phys. 1990;18:455–461. 2105924 10.1016/0360-3016(90)90115-z

[2] Biggs PJ , Russell MD . Effect of a femoral head prosthesis on megavoltage beam radiotherapy. Int J Radiat Oncol Biol Phys. 1988;14:581–586. 3125136 10.1016/0360-3016(88)90279-9

[3] Hazuka MB , Stoud DN , Adams J , Ibbott GS , Kinzie JJ . Prostatic thermoluminescent dosimeter analysis in a patient treated with 18 MV x rays through a prosthetic hip. Int J Radiat Oncol Biol Phys. 1993;14:339–343. 10.1016/0360-3016(93)90358-3 8420884

[4] Hazuka MB , Ibbott GS , Kinzie JJ . Hip prostheses during pelvic irradiation: effect and corrections. Int J Radiat Oncol Biol Phys. 1988;14:1311–1317. 3133330 10.1016/0360-3016(88)90412-9

[5] Burleson WD , Stutzman MT , Stitt J , Karlsson UL , Mian TA . In vivo isocentre dose in two hip prosthesis patients. Int J Radiat Oncol Biol Phys. 1991;20:1347–1352. 2045308 10.1016/0360-3016(91)90248-3

[6] Hudson FR , Crawley MT , Samarasekera M . Radiotherapy treatment planning for patients fitted with prostheses. Br J Radiol. 1984;57:603–608. 6733406 10.1259/0007-1285-57-679-603

[7] Reft C , Alecu R , Das IJ , et al. Dosimetric considerations for patients with HIP prostheses undergoing pelvic irradiation. Report of the AAPM Radiation Therapy Committee Task Group 63. Med Phys. 2003;30:1162. 12852541 10.1118/1.1565113

[8] Moutrie V , Rosenfeld A , Charles P . Prosthesis identification using a megavoltage linac beam Journal of Medical Imaging and Radiology Oncology. 2014;58:207.

[9] Roberts R . How accurate is CT‐based dose calculation on a pencil beam TPS for a patient with a metallic prosthesis. Phys Med Biol. 2001;46:N227–N234. 11580187 10.1088/0031-9155/46/9/402

[10] Gilde‐Hurst C , Chen D , Zhong H , Chetty IJ . Changes realized from extended bit‐depth and metal artifact reduction in CT. Med Phys. 2013;40:061711. 23718590 10.1118/1.4805102

[11] Mutic S , Palta J , Butker EK , et al. Quality assurance for computed tomography simulators and the computed tomography simulation process: report of the AAPM Radiation Therapy Committee Task Group No. 66. Med Phys. 2003;30:2762. 14596315 10.1118/1.1609271

[12] Kairn T , Crowe SB , Kenny J , et al. Dosimetric effects of a high‐density spinal implant. In Journal of Physics: Conference Series 2013 (Vol. 444, No. 1, p. 012108). IOP Publishing. http://iopscience.iop.org/article/10.1088/17426596/444/1/012108/meta.

[13] Palleri F , Baruffaldi F , Angelini AL , Ferri A , Spezi E . Monte Carlo characterization of materials for prosthetic implants and dosimetric validation of Pinnacle 3 TPS. Nuclear Instruments and Methods in Physics Research Section B: Beam Interactions with Materials and Atoms. 2008; 266:5001–5006.

[14] Giantsoudi D , De Man B , Verburg J , et al. Metal artifacts in computed tomography for radiation therapy planning: dosimetric effects and impact of metal artifact reduction. Phys Med Biol. 2017;62:R49–R80. 28323641 10.1088/1361-6560/aa5293

[15] Liugang G , Hongfei S , Xinye N , Mingming F , Zheng C , Tao L . Metal artifact reduction through MVCBCT and kVCT in radiotherapy. Scientific Reports. 2016; 6. 10.1038/srep37608 PMC5116646 27869185

[16] Johns HE , Cunningham JR . The Physics of Radiology, 3rd edn Springfield, IL: Charles C Thomas; 1969.

[17] Hubbell JH . Photon cross sections attenuation coefficients and energy absorption coefficients from 10 keV to 100 GeV. Washington, D.C. 20234: U.S National Bureau of Standards, Centre for Radiation Research; 1969. Report No.: NSRDS‐NBS 29.

[18] Hubble JH , Seltzer SM . Tables of x‐ray mass attenuation coefficients and mass energy‐absorption coefficients. [Online].; 2004 [cited 2017 02 08. Available from: http://physics.nist.gov/xaamdi.

[19] Commissioning and quality assurance of computerized planning systems for radiation treatment of cancer . Technical report series Vienna: International Atomic Energy Agency; 2004. Report No.: 430.

